# Verbal Abilities: Sex Differences in Children at Different Ages

**DOI:** 10.11621/pir.2023.0202

**Published:** 2023-06-15

**Authors:** Irina E. Rzhanova, Olga S. Alekseeva, Anna Ya. Boldyreva, Anastasia Yu. Nikolaeva, Yulia A. Burdukova

**Affiliations:** a Psychological Institute of Russian Academy of Education, Moscow, Russia; b Moscow State University of Psychology & Education, Moscow, Russia

**Keywords:** verbal abilities, sex differences, age differences, intelligence, WISC, WPPSI

## Abstract

**Background:**

The assertion of sex differences in verbal abilities is a highly controversial subject. Some studies have demonstrated a female advantage; other studies have found higher rates in males. The results depended on the type of verbal ability that was studied, the cultural context, and the ages of the subjects. There are two types of theories that have been developed to explain the existence of sex differences in cognitive abilities. Social theories explain the differences as caused by social determinants. Biological theories consider biological factors such as prenatal development conditions and hormone levels, among others, as the cause of sex differences.

**Objective:**

To investigate sex differences in verbal abilities in children of different ages.

**Design:**

Two different editions of Wechsler tests were used. For children age 2.5 to 5 years, the Wechsler Preschool Primary Scale of Intelligence (WPPSI-IV) was used. For children age 6 and older, we administered the Wechsler Intelligence Scale for Children (WISC-V). The total sample included 313 children.

**Results:**

The study found significant sex differences in performance on the Verbal Comprehension Scale in children of different ages. At the age of 2 to 4 years, the girls performed better than the boys. In the group of boys, there was a significant increase in verbal abilities at the age of 8-9 years. By the age of 10-11 years, boys began outperforming girls on the Verbal Comprehension Index. Scores on the Verbal Comprehension and Visual Spatial subtests for the boy sample showed stronger correlations than in the girl sample in all age groups.

**Conclusion:**

Sex differences in verbal abilities varied depending on the age of the children. The boys showed a stronger integration of their verbal abilities into the structure of their intelligence than the girls.

## Introduction

### Sex differences in verbal abilities

Verbal abilities can be defined as the ability to analyze language-based information and solve verbal reasoning and inference problems. Sex differences in verbal abilities have been the focus of many studies in psychology; however, their results have turned out to be rather contradictory. Some studies found an advantage for females in this type of ability (Shcheblanova, 2008; Stevenson & Newman, 1986; Strand et al., 2006; Barel & Tzischinsky, 2018; Hirnstein et al., 2023); others found higher rates in males (Hogrebe et al., 1985; Jensen & Reynolds, 1983; Liu & Linn, 2015; Pezzutti & Orsini, 2016; Sokolowski et al., 2020); and still other studies did not find any sex differences in verbal abilities at all (Rzhanova & Alekseeva, 2016; Cole & LaVoie, 1985; Lynn et al., 2005).

Its important to stress that all these results depended on what type of verbal ability was being studied. According to meta-analysis data, one can see that girls performed better in general verbal ability, vocabulary, and anagramming, while boys outperformed on verbal analogies (Hyde & Lynn, 1988). The ability of males to solve verbal analogies better than females was later confirmed in other studies (Colom et al., 2004). In a recent meta-analysis involving about 350,000 participants, girls/ women outperformed boys/men in phonemic fluency, recall, and recognition. The dependence of the effect on the region and language of the subjects was also established (Hirnstein et al., 2023).

Sex differences in verbal abilities often rely on cultural differences. Thus, data obtained in one country could easily differ from similar studies conducted in another country. A study involving Italian children age 6 to 16 using the WISC-V (Pezzutti & Orsini, 2016), demonstrated an advantage for boys over girls on the Verbal Comprehension index (Similarity and Vocabulary subtests). In a sample of Taiwanese preschoolers, boys significantly outperformed girls on verbal WPPSI-IV subtests such as Information, Vocabulary, Comprehension, and Picture Naming (Chen & Lynn, 2021). At the same time, however, a U.K.-based study of children age 11 to 12 demonstrated that girls scored significantly higher than boys on the Verbal Reasoning of Cognitive Abilities Test index. The results revealed an advantage in all three subtests: Verbal classification, Sentence completion, and Verbal analogies (Strand et al., 2006).

The superiority of girls over boys in the U.K. sample was associated with the existence of gender equality policies in their schools. Gender equality policy has been practiced in educational institutions in the United Kingdom since the mid-1970s (Shcheblanova, 2008). The connection of sex differences in cognition with the policy of gender equality is supported by data from the French standardization of the four Wechsler scales from 1981 to 2016. Boys scored higher on the three WISC-III verbal subtests, but sex differences became insignificant on the WISC-IV and WISC-V verbal subtests in the 2000s (Grégoire, 2020).

Differences in verbal abilities may depend on general intelligence. This conclusion was reached on a sample of Russian children (Saulina, 2015). The study demonstrated that in the group of children with IQ scores above 115, girls coped significantly better with verbal tests than boys did (in the Missing Words and Analogies tests). Meanwhile, in the sample of children with lower IQ scores, boys and girls demonstrated equal results in language abilities.

Apparently, age plays an important role in the influence of sex on verbal abilities. As we saw in previous studies, differences in verbal abilities between boys and girls depended on the age group they belonged to. A twin longitudinal study of sex differences in childhood and adolescence has shown that girls were ahead of boys in verbal ability tests at the ages of 2 to 4. Boys had significantly higher scores at ages 10 and 12. No sex differences were found in other age groups (Toivainen et al., 2017). A study of gifted children made by Shcheblanova on a Russian-speaking sample found no gender differences in elementary school children; in the fifth or sixth grade, boys began to outperform girls, but in the seventh grade, the differences were again erased (Shcheblanova, 2008).

Sex differences were examined in a study of Russian-speaking fourth graders at ages 10 and 11. Girls outperformed boys in all analyzed parameters: reading speed, the score on the Russian Language Achievement Test, and final grades at school. It is interesting that, in the boys’ sub sample, non-verbal intellect was a significant predictor of two indicators: success in the final Russian language test and reading skills (Tikhomirova et al., 2020).

### Theories of sex differences in cognitive abilities

There are two types of theories which have been developed to explain the existence of sex differences in cognitive abilities. The first group explains the differences between boys and girls by social determinants. For example, parents and teachers are subjectively inclined to evaluate boys as more capable. Teachers often pay more attention to boys than girls in their classes (Shcheblanova, 2008). The second group of theories can be called biological, because they consider biological factors to be the cause for sex differences in verbal ability (Hirnstein et al., 2019). There are well-known studies of the effects of prenatal testosterone levels on cognitive abilities, particularly visuospatial abilities (Puts et al., 2008; Heil et al., 2011). Testosterone levels in the mother’s amniotic fluid can influence the child’s verbal abilities. Lutchmaya’s study showed the effect of amniotic fluid testosterone levels on the development of verbal abilities of children between 1 and 2 years of age. A high hormone level was negatively associated with vocabulary size at this age (Lutchmaya et al., 2001).

Thus, one can see many factors influencing general language abilities: the age of respondents, their cultural background, and the type of specific language skills explored in the study, as well as the methods used in the study. The objective of our study was to investigate sex differences in verbal abilities at different age groups. We posed the following research questions:

1. How do sex differences in verbal abilities change depending on children’s age?
2. How are verbal abilities related to general intelligence in different age groups of boys and girls?

## Methods

### Participants

A total sample of 313 children took part in our study. All the children had been attending the public kindergartens and schools in Moscow region. Informed consent was obtained from parents before the study. The data was collected from 2017 to 2020. Participants were divided into five groups depending on their age. The characteristics of each group are presented in *Table 1*.

**Table 1:** Age characteristics of participants

	N	M(years)	SD	female/male (%)
Group 1 (2.5-4 years)	84	3.5	0.3	45/55
Group 2 (4-5 years)	56	4.6	0.4	39/61
Group 3 (6-7 years)	64	6.4	0.4	58/42
Group 4 (8-9 years)	45	8.2	0.6	53/47
Group 5(10-11 years)	64	10.6	0.3	42/58

### Procedure

Two different editions of Wechsler tests were used to assess the children's cognitive abilities.

### Wechsler Preschool and Primary Scale of Intelligence (WPPSI-IV)

For children age 2.5 to 5 years, the Wechsler Preschool and Primary Scale of Intelligence (WPPSI-IV) was used (see psychometric properties in Wechsler, 2012). In the age group from 2.5 to 4 years, this test has three integral scales: the Verbal Comprehension Index, the Working Memory Index, and the Visual Spatial Index. The Verbal Comprehension Index includes the Passive Vocabulary and Information subtests.

For the age of 4 to 5 years, the WPPSI-IV test includes five integral scales: the Verbal Comprehension Index, the Visual Spatial Index, the Fluid Intelligence Index, the Working Memory Index, and the Processing Speed Index. The Verbal Comprehension Index consists of the Information and Similarities subtests. The structure of the test on Russian preschoolers has been studied previously (Rzhanova et al., 2018).

### Wechsler Intelligence Scale for Children (WISC-V)

For children 6 years of age and older, we used the Wechsler Intelligence Scale for Children WISC-V (see psychometric properties in Wechsler, 2014). This test also contains five integral scales: the Verbal Comprehension Index, the Visual Spatial Index, the Fluid Intelligence Index, the Working Memory Index, and the Processing Speed Index. The Verbal Comprehension Index includes Similarities and Vocabulary subtests.

Pearson's correlation coefficient and Dispersion Analyzes (ANOVA) were used to process the data.

## Results

### Verbal Comprehension Index: effects of age and sex

**Figure 1.**
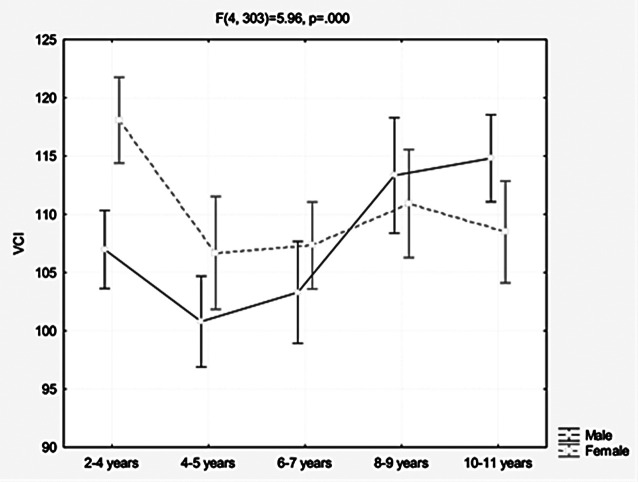
Verbal Comprehension Index: age and sex differences

The age dynamics of the Verbal Comprehension Index in the male and female samples are shown in *Figure 1*. Girls scored higher than boys in the younger age group (F = 19.26, p = .00), but then the results in the girl sample decreased (F= 13.59, p = .00); after this drop, they remained at approximately the same level. In the group of boys there was a significant increase in verbal abilities at the age of 8-9 years (F = 8.94, p = .00); by the age of 10-11 years, boys began overtaking girls on the Verbal Comprehension Index (F = 4.70, p = .03).

In the girl sample we could see a decrease in the Verbal Comprehension score. This drop is observed from the younger preschool age to the middle preschool age. Most likely it could be attributed to different methods of diagnosing verbal comprehension abilities. At the younger age of 4-5 years, two subtests were used to evaluate general verbal abilities: Passive Vocabulary and Information. For children older than 6 years, another pair of subtests was used: Information and Similarities. It can be assumed that the Passive Vocabulary subtest is significantly easier than the Similarities subtest, so this could explain why girls in the younger group would obtain higher scores.

### Comparison of group data for Verbal Comprehension subtests

We then looked at the sex differences on the Passive Vocabulary subtest. According to our data, in the 2-4 year old age group, girls scored significantly better than boys (F = 17.08, p = .000). Sex differences for the Information subtest were observed only in the younger age group of 2-4 years old (F = 8.72, p = .000); the girls scored higher. However, we did not find any sex differences in the scores of the older group.

**Figure 2.**
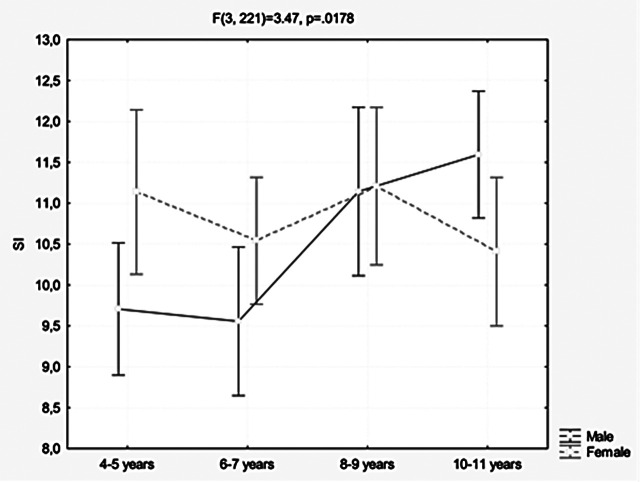
Similarity subtest: age and sex differences

*Figure 2* shows the scores on the Similarity subtest. In general, the data obtained in this subtest goes in line with the data for the general Verbal Comprehension Index. At a younger age (4-5 years), the results revealed a clear female advantage (F = 4.77, p = .003). At 6-9 years the differences were insignificant, and starting from 10 years, they began to reach a significant level but in the opposite direction: boys outperformed girls (F = 3.85, p = .05). Moreover, boys significantly improved their results at the age of 8-9 years compared with younger ages (F = 5.20, p = .002).

There were no significant differences between the groups of boys and girls on the Vocabulary subtest scores. However, boys significantly improved their performance at the age of 8-9 years (F = 8.93, p = .00).

### The relationship of verbal abilities and general intelligence depending on age and sex

**Figure 3.**
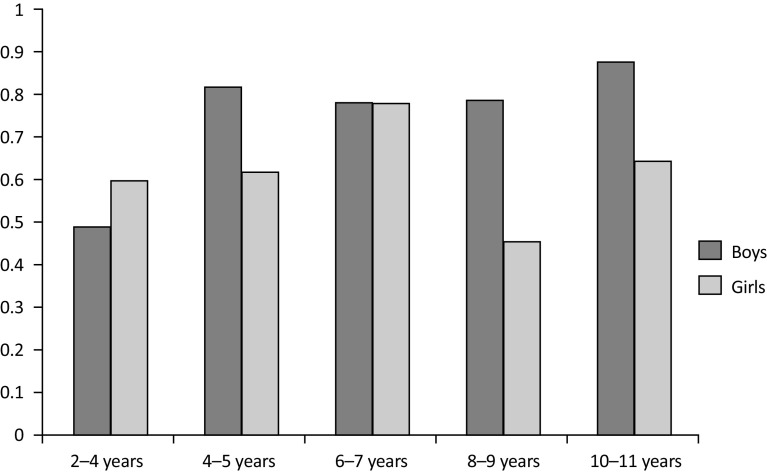
Correlations between the verbal comprehension index and the total score in the groups of boys and girls.

We analyzed the connections between verbal ability and general intelligence. *Figure 3* shows the correlation coefficients on the Verbal Comprehension Index and the Full-Scale Intelligence Quotient. All these relationships were significant. Moreover, in the older groups (from 8-years-old on) higher correlations were observed between verbal ability and general intelligence in the group of boys compared to the group

The relationships between the verbal scale and other main integral scales of the Wechsler tests were investigated (see *Table 2)*. Results on the Verbal subtests and Visual Spatial subtests in the male sample showed stronger correlations than in the female sample in all age groups. Similar results were obtained for the scores on the Fluid Intelligence Index: the correlations of those scores with the general verbal abilities in the male sample were higher than in the female sample (except for the age group of 4-5 years). The most obvious differences in the pattern of relationships were observed in the oldest group, where the correlations between verbal abilities and all other cognitive characteristics were higher for the boys.

**Table 2:** Correlations of the Verbal Comprehension Index scores with other cognitive scales in the samples of boys and girls

	VSI		WMI		FRI		PSI	
	Boys	Girls	Boys	Girls	Boys	Girls	Boys	Girls
2-4 years	.38*	.31	.28	.29				
4-5 years	59**	-.10	.35	-.36	.17	.22	.37	-.19
6-7 years	.43*	.42*	.57**	.71**	.46*	.35	.31	.39*
8-9 years	.31	.23	.56**	.64**	.59**	.26	.27	.15
10-11 years	.62**	.07	.64**	.44*	.52"	.33	.49*	.33

*p < 0.05; **p < 0.01

## Discussion

The assertion of sex differences in verbal abilities is highly controversial. Some studies have demonstrated a female advantage (Strand et al., 2006; Hirnstein et al., 2023; Reilly et al., 2019; Peterson, 2018); other studies have found higher rates in men (Pezzutti & Orsini, 2016; Sokolowski et al., 2020; Lynn, 2021).

The current study investigated sex and age differences in verbal abilities and their associations with other cognitive abilities. Analysis of different age groups revealed that at the younger preschool age, the girls coped better with verbal ability tasks than the boys did. These findings are consistent with a lot of early research (Reilly et al., 2019; Peterson, 2018). Then, at older ages, the differences began to disappear, and by the age of 8-9 years, boys significantly improved their performance, while the results in the female sample remained at the same level. The differences became significant in favor of boys by the age of 10-11 years.

Apparently, the critical point for the development of verbal abilities in the male sample was their beginning school education (in the current sample, children age 6-7 have not yet attended school). Similar results were obtained in a twin longitudinal study by Toivainen (Toivainen et al., 2017). In this study girls outperformed boys in general verbal abilities during the early preschool years. However, by the age of 11 years, boys significantly outperformed girls (Toivainen et al., 2017). It is difficult to declare without additional research what exactly influences the success boys achieve with the beginning of schooling: these can be due to both physiological features (the next stage in the maturation of brain areas involved in speech production) and social factors (a preference for boys by both parents and teachers). It should also be noted that education in Russian schools is based mainly on verbal activities, and it is possible that the verbal abilities of boys at primary school age are more sensitive to environmental influences than those of girls.

The Similarities subtest allows us to estimate the development of children's conceptual thinking and their ability to generalize and classify verbal material, as well as their ability to compare and organize information (Wechsler, 2014). At the preschool age, girls performed better on this subtest than boys did; at primary school age, the sex differences were insignificant, but starting from the age of 10, they began to reach a level of significance in favor of boys. For the rest of the verbal comprehension subtests no significant sex differences were found. However, data analysis of the Similarities subtest produced very similar results to the score level obtained in the analysis of the overall Verbal Comprehension Index score. This suggests the Similarities subtest is the key one in assessing differences in verbal ability.

Our data revealed sex differences for the relationship between general Verbal Comprehension and other cognitive abilities. A clear trend can be seen for verbal abilities. The relationship between verbal ability and general intelligence and other cognitive abilities increases with age in boys, while the correlation begins to weaken in girls. At primary school age, boys’ verbal ability is more cognitively based than that of girls. Similar results were obtained earlier on a sample of fourth graders: the success of boys in completing tasks in the Russian language turned out to be more associated with non-verbal intelligence compared to girls (Tikhomirova et al., 2020).

## Conclusion

The present study revealed the dependence of verbal intelligence scores, as assessed by the Wechsler test, on age and sex. At a younger preschool age, girls got better results on the Verbal Comprehension Index than boys. The differences became significant in favor of boys by the age of 10-11 years. The study found sex differences for the correlations between Verbal Comprehension and other cognitive abilities. The relationship between verbal ability and other abilities increased with age in boys, while it began to weaken in girls. Further work will be required to conduct a longitudinal study in order to examine the development of verbal abilities in childhood.

## Limitations

The limitations of the study are related to the available data. Although the children participating in the study lived in the same language region with the same educational traditions, we collected no data on their socio-demographic status and the types of schools and kindergartens they attended; therefore, it is not possible to conclude that the cohort samples were equal.
